# Endoglucanase 2 (Eng2), a shared immunodominant antigen in dimorphic fungi that elicits immunity during infection

**DOI:** 10.1172/JCI191103

**Published:** 2025-09-25

**Authors:** Uju J. Okaa, Cleison Ledesma Taira, Lucas dos Santos Dias, Hannah Dobson, Gregory C. Kujoth, Althea Campuzano, E. Jane Homan, George R. Thompson, Chiung-Yu Hung, George S. Deepe, Marcel Wüthrich, Bruce S. Klein

**Affiliations:** 1 Departments of Pediatrics, University of Wisconsin–Madison (UW-Madison), Madison, Wisconsin, USA.; 2 Fungal Pathogenesis Section, Laboratory of Clinical Immunology and Microbiology, and; 3 Molecular Mycology and Immunity Unit, Laboratory of Host Immunity and Microbiome, National Institute of Allergy and Infectious Diseases (NIAID), NIH, Bethesda, Maryland, USA.; 4 General Dynamics Information Technologies, The Air Force Research Laboratory, Fort Sam Houston, Texas, USA.; 5 ioGenetics LLC, Madison, Wisconsin, USA.; 6 Department of Internal Medicine, University of California Davis Medical Center, Sacramento, California, USA.; 7 Department of Medicine, Division of Infectious Diseases, University of Cincinnati College of Medicine, Cincinnati, Ohio, USA.; 8 Internal Medicine, and; 9 Medical Microbiology and Immunology, University of Wisconsin School of Medicine and Public Health, UW-Madison, Madison, Wisconsin, USA.

**Keywords:** Immunology, Infectious disease, Adaptive immunity, Antigen, T cells

## Abstract

We describe here a shared surface and cell wall protein, endoglucanase 2 (Eng2), expressed on the etiological agents that cause the endemic systemic mycoses of North America — *Blastomyces*, *Coccidioide*s, and *Histoplasma*. We demonstrate that, despite sequence variation of the protein across these related fungi, exposure to Eng2 vaccinated and protected inbred and humanized HLA-DR4 strains of mice against lethal experimental infections with these fungi by eliciting adaptive immunity mediated by CD4^+^ T cells. We also show that CD4^+^ T cell precursors against Eng2 were detectable in naive individuals and that patients who had recovered from these infections evinced a memory and recall CD4^+^ T cell response to Eng2 and its immunodominant epitopes that we have mapped. We created and cataloged new tools and information, such as immunodominant peptide epitopes of Eng2 from each fungus recognized by inbred mice and humans, and we engineered peptide–MHC II tetramers to track T cells in inbred and HLA-DR4–humanized mice. These tools and tetramers will be useful for those who study these infections in mice and humans. Last, because most patients demonstrated immune memory and recall responses against Eng2, our work offers tools for the diagnosis of this collection of infectious diseases across North America.

## Abstract 2

We identified a major antigen expressed in a family fungal infectious diseases common in North America. The antigen stimulates potent immunity in mice and humans.

## Introduction

The endemic dimorphic fungi of North America including *Blastomyces*, *Coccidioides*, and *Histoplasma* collectively cause over a million new infections each year and represent the most common cause of fungal pneumonia in otherwise healthy individuals. Many of these infections are mild or asymptomatic, but increasing numbers represent reactivation or new acquisition of severe disease in the setting of biologics and other forms of immune suppression. The endemic zones of these pathogens also are spreading, probably due to anthropurgic behavior and climate.

Vaccination against infectious diseases has substantially improved public health. Once-common and deadly bacterial and viral diseases are now rare or eliminated in vaccinated populations (1). Despite these successes and the growing clinical need, there are currently no fungal vaccines licensed for human use (2). A major limitation for developing vaccines against fungi stems from the lack of knowledge of highly protective antigens, especially those that are shared and can protect against multiple fungi. Such immunodominant antigens could also be used for developing better methods of diagnosis.

We recently discovered *Blastomyces* endoglucanase 2 (*Bl*-Eng2), a glycoprotein in *Blastomyces dermatitidis* that harbors potent antigenic (3) and adjuvant properties (4). *Bl*-Eng2 elicits cellular immunity and protects against experimental infection with this fungus. A peptide-MHC tetramer specific to an immunodominant region of the antigen demonstrated that vaccination and infection expanded and recruited several hundred thousand T cells to the lung after infection (3). To our knowledge, this is the largest activation and expansion of functional antifungal T cells reported to date. The potency and functional versatility of this antigen and its similarity across the dimorphic fungi prompted us to investigate whether vaccination with *Bl*-Eng2 or its homologs could protect against infection with the other dimorphic fungi of North America and thus serve as a pan-fungal antigen for vaccination and diagnostics.

Here, we report that vaccination with Eng2 homologs protected against experimental infection with corresponding dimorphic fungi in inbred C57BL/6 mice and humanized HLA-DR4 mice. We also show that CD4^+^ T cell precursors against these antigens were detectable in the blood of infection-naive participants and observed that patients who had recovered from each of these infections demonstrated a memory or recall CD4^+^ T cell response to the antigen. Given the immunological relevance of Eng2, we mapped the immunogenic T cell epitopes. Importantly, we have now created and cataloged a compendium of new epitope information and related immunological tools such as the immunodominant peptides of the Eng2 homolog in each dimorphic fungus for both strains of mice and study participants, as well as peptide–MHC II tetramers for tracking CD4^+^ T cells in inbred C57BL/6 and HLA-DR4 humanized mice. This new information and the accompanying tools will be invaluable for large numbers of investigators who study basic and clinical questions about immunity to these infections in mice and humans. Last, as most patients demonstrate memory immunity and recall responses against Eng2, our work offers tools for the diagnosis of this collection of important infectious diseases across North America.

## Results

### Eng2 homologs are shared and associated with the cell wall of dimorphic fungi.

**Figure 1:**
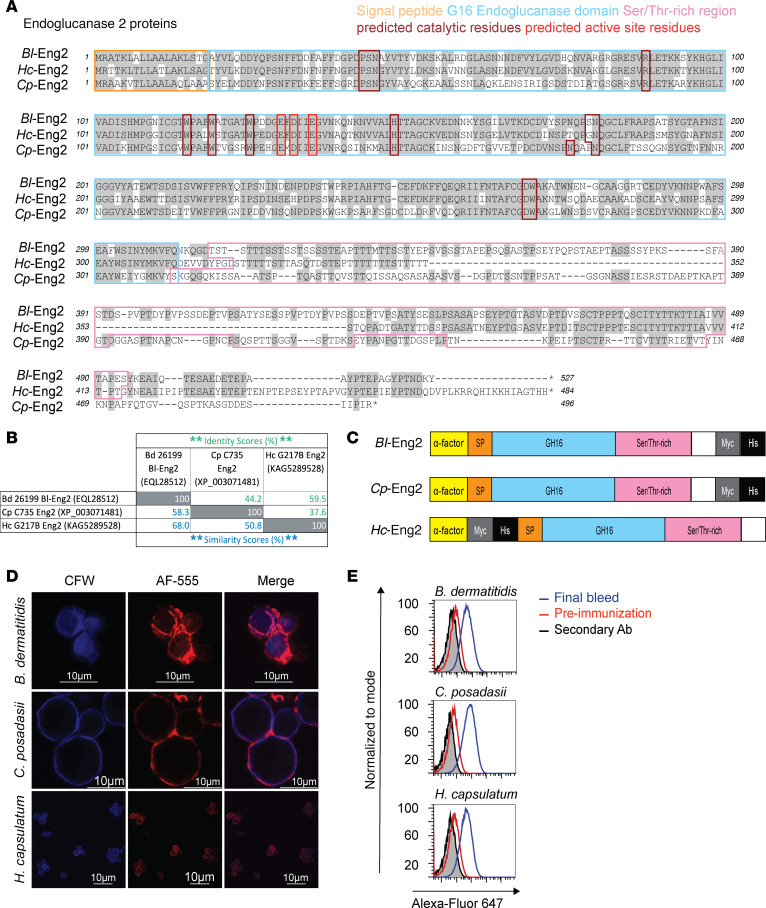
Alignment of Eng2 aa sequences of dimorphic fungi. (**A**) Amino acid sequences of the Eng2 homologs. Sequences were aligned using the T-Coffee algorithm. The GH16 endoglucanase domains and associated predicted active sites and catalytic residues were annotated by the NCBI Conserved Domain Database. The serine/threonine-rich domains were calculated as in González et al. (36) (with parameter density = 40%, limit = 8, window = 20, separator = 5). The outlined regions are as follows: orange, native signal peptide; blue, region homologous to GH16 subfamily 1; maroon, predicted active site residues; red, predicted catalytic residues; pink, serine/threonine-rich domain. (**B**) The percentage of identical and similar aa between Eng2 homologs was determined by T-Coffee multiple sequence alignment. (**C**) Domains of recombinant Eng2 proteins: yellow, α-factor secretory signal; SP, signal peptide (orange), native Eng2 signal peptide; blue, GH16 domain; pink, serine/threonine-rich domain; gray, c-Myc tag; and black, histidine tag. (**D**) Immunofluorescence microscopy images showing cell wall localization of Eng2 in *B*. *dermatitidis* yeast, *C*. *posadasii* spherules, and *H*c yeast. CFW, Calcofluor White staining of fungal chitin; AF-555, Alexa Fluor 555 rabbit polyclonal anti-Eng2 antibody raised against each homolog. Scale bars: 10 μm. (**E**) Flow cytometric analysis of *B*. *dermatitidis* and *H*. *capsulatum* yeast and *C*. *posadasii* spherules stained with rabbit preimmune control serum and anti-Eng2 rabbit immune serum. “Normalized to mode” refers to a *y*-axis scaling option for histograms that normalizes each peak to its highest point, making all peaks appear at the same height, even if the populations have different numbers of events. This is helpful for visualizing and comparing populations with varying event counts.

Subcutaneous vaccination with *Bl*-Eng2 protects mice against experimental infection with *B*. *dermatitidis* (3). We found that the aa sequences for the respective Eng2 homologs of the 3 dimorphic fungi *B*. *dermatitidis* (*Bl*-Eng2), *Coccidioides posadasii (Cp*-Eng2), and *Histoplasma capsulatum* (*Hc*-Eng2) was shared (Figure 1A). The sequence similarity for the 3 homologs ranged from 50%–68%, and the identity ranged from 38%–60% (Figure 1B). Given these results, we sought to test whether vaccination with *Bl*-Eng2 also protects mice against infection with *C*. *possadasii* and *H*. *capsulatum*. We found that lung CFU of *Bl*-Eng2–vaccinated mice were similar to those of unvaccinated mice after infection with *C*. *possadasii* or *H*. *capsulatum* (Supplemental Figure 1; supplemental material available online with this article; https://doi.org/10.1172/JCI191103DS1). Thus, despite the sequence similarity and identity of Eng2 across these dimorphic fungi, vaccination with *Bl*-Eng2 did not cross-protect against experimental infection with *C*. *posadasii* or *H*. *capsulatum*. We thus cloned the homologs of the respective dimorphic fungi, expressed them in *Pichia pastoris* (Figure 1C), and raised antibodies against each protein homolog. Staining of yeast with these antibodies and analysis by fluorescence microscopy and FACS localized Eng2 homologs to the cell wall (Figure 1, D and E).

### Protective efficacy of Eng2 homologs against endemic dimorphic fungi.

**Figure 2:**
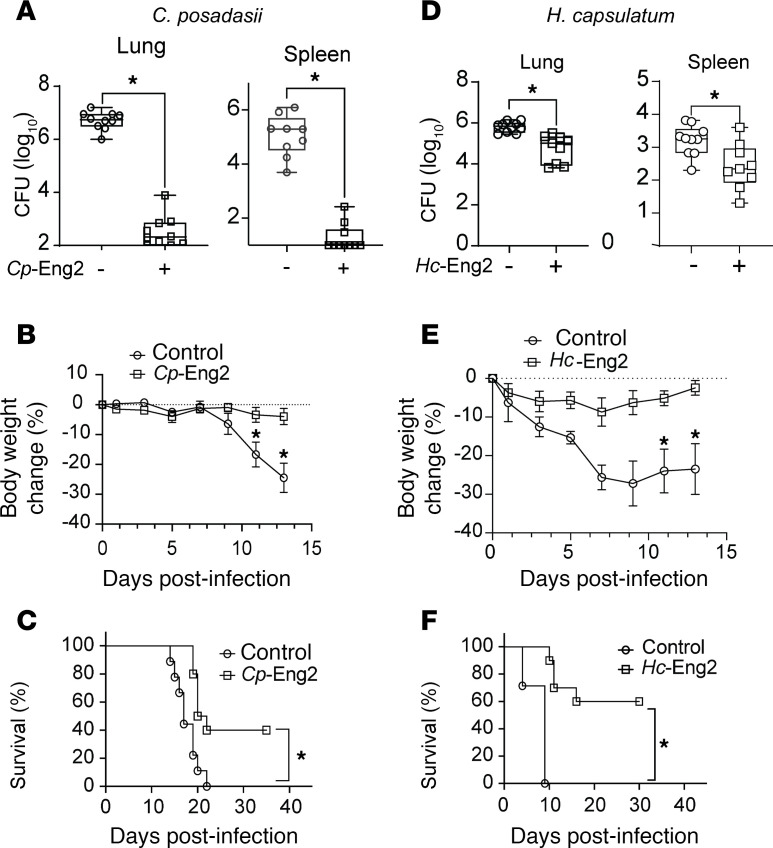
Protective efficacy of Eng2 against endemic dimorphic fungi. The homologous Eng2 proteins *Cp*-Eng2 and *Hc*-Eng2 were respectively used to vaccinate C57BL/6 mice against *C*. *posadasii* (**A**–**C**) and *H*. *capsulatum* (**D**–**F**), as described in Methods. Burden of infection (measured in CFU), weight loss, and survival (from top to bottom). Percentage of body weight change is depicted for mice monitored daily. Data shown are from a representative experiment of 2–3 experiments (*n* = 10 mice/group). CFU are expressed as log_10_ and plotted using box-and-whisker plots showing the median as well as the minimum and maximum values of all the data as noted in Methods. *P < 0.05, by 2-tailed Mann-Whitney *t* test. Other data are mean ± SD.

To investigate whether vaccination with *Cp*-Eng2 or *Hc*-Eng2 protects mice against respective infection with *C*. *posadasii* and *H*. *capsulatum*, we purified recombinant proteins and generally loaded them into glucan-chitin particles (GCPs) as described before (5). Vaccination with *Cp*-Eng2 followed by infection with *C*. *posadasii* reduced CFU in the lung and spleen by 3–4 logs compared with unvaccinated controls (Figure 2 A). As a consequence of increased resistance, vaccinated mice did not lose weight, unlike the unvaccinated mice (Figure 2B), and showed an increased survival rate following lethal pulmonary infection compared with unvaccinated control mice (Figure 2C). Similarly, vaccination with *Hc*-Eng2 protected C57BL6 mice against *H*. *capsulatum* following lethal pulmonary infection (Figure 2, D–F).

### Identification of immunogenic CD4^+^ T cell epitopes of Cp-Eng2 and Hc-Eng2 in C57BL6 mice.

**Figure 3:**
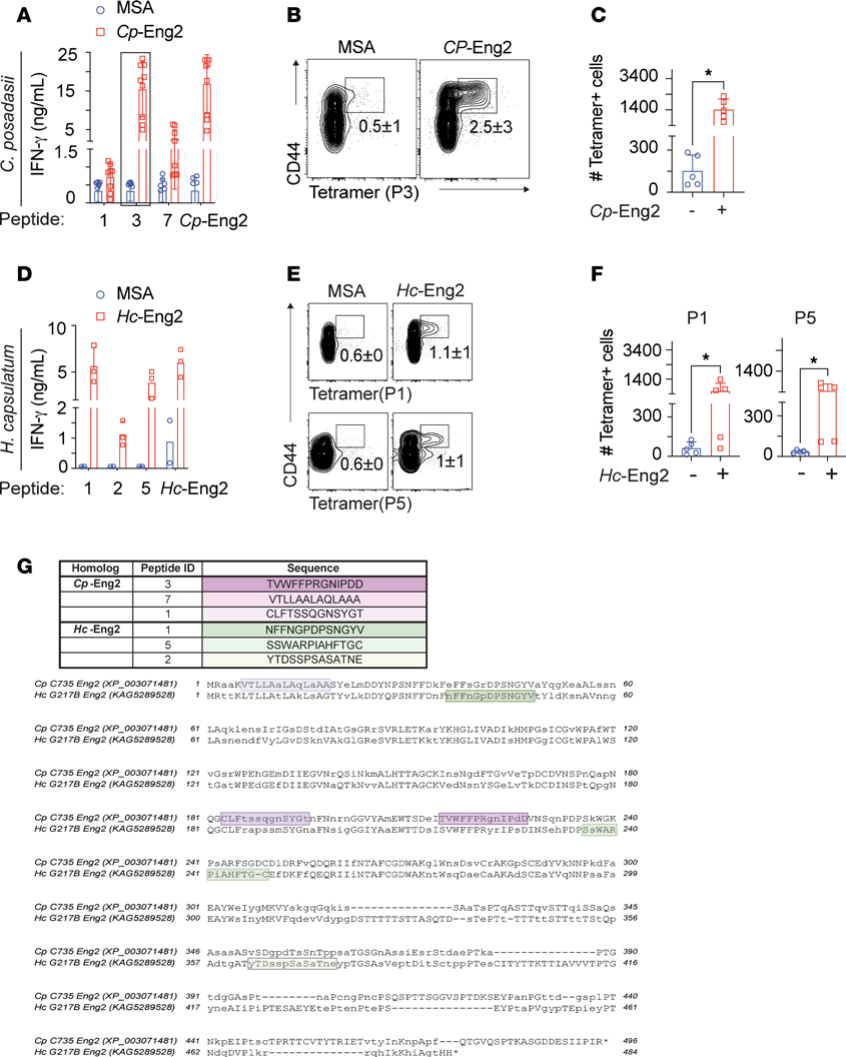
Mapping of Eng2 immunodominant epitopes in *C*. *posadasii* and *H*. *capsulatum* in C57BL/6 mice. (**A** and **D**) Identification of immunodominant epitopes of *Cp*-Eng2 and *Hc*-Eng2. Mice (*n* = 5/group) were vaccinated with *Cp*-Eng2 or *Hc*-Eng2 as described in Methods. Two weeks after the boost, splenocytes were restimulated ex vivo with peptide P3 from *Cp*-Eng2 (**C** and **D**) or P1 and P5 from *Hc*-Eng2. (**B**, **C**, **E**, and **F**) Splenocytes from vaccinated mice were stained with tetramers containing peptide P3 (*Cp*-Eng2), P1 (*Hc*-Eng2), or P5 (*Hc*-Eng2) and analyzed by flow cytometry. The frequency and number of tetramer^+^ cells are illustrated in **B**, **C**, **E**, and **F**. The data shown are representative of 2 independent experiments and are presented as the mean ± SD. *P < 0.05, by 2-tailed Mann-Whitney *U* test. (**G**) *Cp*-Eng2 and *Hc*-Eng2 sequences were aligned with the T-Coffee algorithm using MacVector 18.5.1. Experimentally determined immunodominant epitopes are highlighted in purple and green gradient-shaded boxes for *Cp*-Eng2 and *Hc*-Eng2, respectively. Peptides that elicited the most IFN-γ (*Cp*-P3 and *Hc*-P1) are shaded dark purple or green.

Since vaccine protection required immunization with homologous Eng2 proteins, we hypothesized that the immunogenic T cell epitopes are different for each homolog. To test this idea, we mapped the Eng2 peptide epitopes recognized by CD4^+^ T cells and generated class II MHC–peptide tetramers as described previously (5, 6). We analyzed the *Cp*-Eng2 and *Hc*-Eng2 homologs for MHC class II peptide–binding sequences. Of 7 predicted peptide epitopes from these homolog, three 13 mers from each one triggered IFN-γ production by CD4^+^ T cells isolated from the spleens of mice vaccinated with the corresponding Eng2 protein (Figure 3, A and D). Peptide 3 from *Cp*-Eng2 (TVWFFPRGNIPDD) and peptides 1 and 5 from *Hc*-Eng2 (NFFNGPDPSNGYV and SSWARPIAHFTGC) induced the strongest IFN-γ response (Figure 3, A, D, and G). Thus, mapping the position of the immunogenic *Cp*-Eng2 and *Hc*-Eng2 peptides indicated different locations of the immunodominant CD4^+^ T cell epitopes in each homolog (Figure 3G). These regions also differed from that in *Bl*-Eng2 (data not shown) (3).

We used the immunodominant peptides to create MHC class II tetramers and to demonstrate expansion of primed Eng2 antigen–specific memory CD4^+^ T cells in the spleens of vaccinated mice (Figure 3, B, C, E, and F). Approximately 12% of CD4^+^ T cells stained with the tetramer and the CD44 activation marker after vaccination with *Cp*-Eng2 and approximately 3% of CD4^+^ T cells did so following vaccination with *Hc*-Eng2. We validated that tetramer binding was specific, since few CD8^+^ T cells bound to the respective tetramers (Supplemental Figure 2A). The precursor frequency of naive (CD44^lo^) Eng2-specific CD4^+^ T cells in unvaccinated mice was 32 for *Hc*-Eng2, 97 for *Bl*-Eng2, and 130 for *Cp*-Eng2) (Supplemental Figure 2C). Vaccination with the corresponding Eng2-homologs activated and expanded tetramer^+^ CD4^+^ T cells by 7- to 29-fold compared with the numbers present in naive mice (Supplemental Figure 2, B and C). In sum, mapping the position of the immunogenic *Cp*-Eng2 and *Hc*-Eng2 peptides indicated different locations of the T cell epitopes (Figure 3G). Thus, we have generated tetramers against all 3 homologs of Eng2 proteins, which allowed tracking and enumeration of Eng2-specific CD4^+^ T cells during the evolution of immunity against these endemic systemic mycoses.

### Functional analysis of Eng2-specific CD4^+^ T cells in vaccinated C57BL/6 mice following infection with C. posadasii and H. capsulatum.

**Figure 4:**
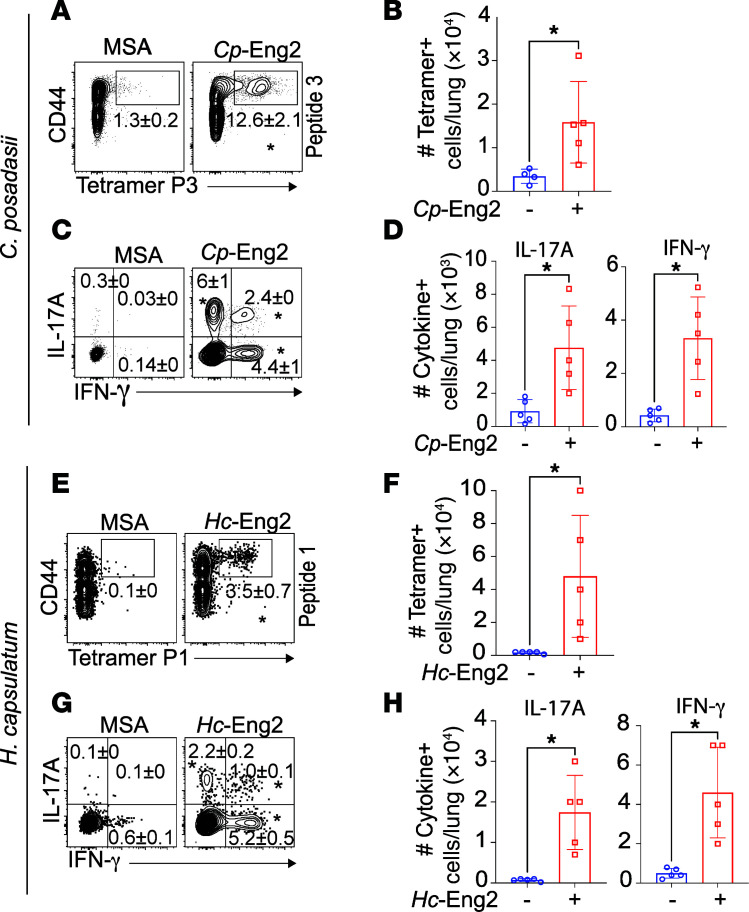
Functional analysis of Eng2-specific CD4^+^ T cells following experimental pulmonary infection with *C*. *posadasii* and *H*. *capsulatum*. Tetramer^+^ cells were determined after C57BL/6 mice were vaccinated as described in Methods with *Cp*-Eng2 (**A**–**D**) or *Hc*-Eng2 (**E**–**H**) (*n* = 5/group). Mice were experimentally infected as in methods and sacrificed 4 or 5 days after challenge followed by enumeration of CD4^+^ tetramer^+^ T cells in the lungs. **A** and **E** show contour flow plots with the percentages of tetramer^+^ CD4^+^ T cells; **B** and **F** show histogram bar graphs with the total number of tetramer^+^ cells. After intracellular staining of IFN-γ and IL-17, the frequencies (**C** and **E**) and numbers (**D** and **H**) of cytokine-producing cells were analyzed. Data are represented as the mean ± SD. *P < 0.05 versus the control group; data were analyzed with the 2-tailed Mann-Whitney *U* test.

We previously reported that *Bl*-Eng2–specific CD4^+^ T cells are recalled to the lungs in substantial numbers upon infection in vaccinated C57BL/6 mice (3). Here, we investigated whether *Cp*- and *Hc*-Eng2–specific CD4^+^ T cells also accumulate in the lungs in substantial numbers in vaccinated mice infected with either fungus. The frequencies and numbers of activated (CD44^+^) tetramer^+^ T cells were at least 10 times higher in vaccinated mice compared with unvaccinated mice at post-infection day 5 or 6 after infection with *H*. *capsulatum* or *C*. *posadasii*, respectively (Figure 4, A, B, E, and F). Since vaccine immunity to dimorphic fungi is primarily mediated by Th1 and Th17 cells that respectively produce IFN-γ and IL-17 (5, 7–11), we stimulated primed T cells with the peptides that were used to generate the tetramers and measured cytokine production by intracellular staining. The frequencies and numbers of IFN-γ– and IL-17-producing T cells from vaccinated mice were significantly increased compared with unvaccinated controls and indicated a mixed Th1/Th17 phenotype (Figure 4, C, D, G, and H). Thus, the tetramer^+^ cells were functional with respect to cytokine production.

### Vaccine protection is conferred by immunodominant peptides versus full-length Eng2 proteins in C57BL/6 mice.

**Figure 5:**
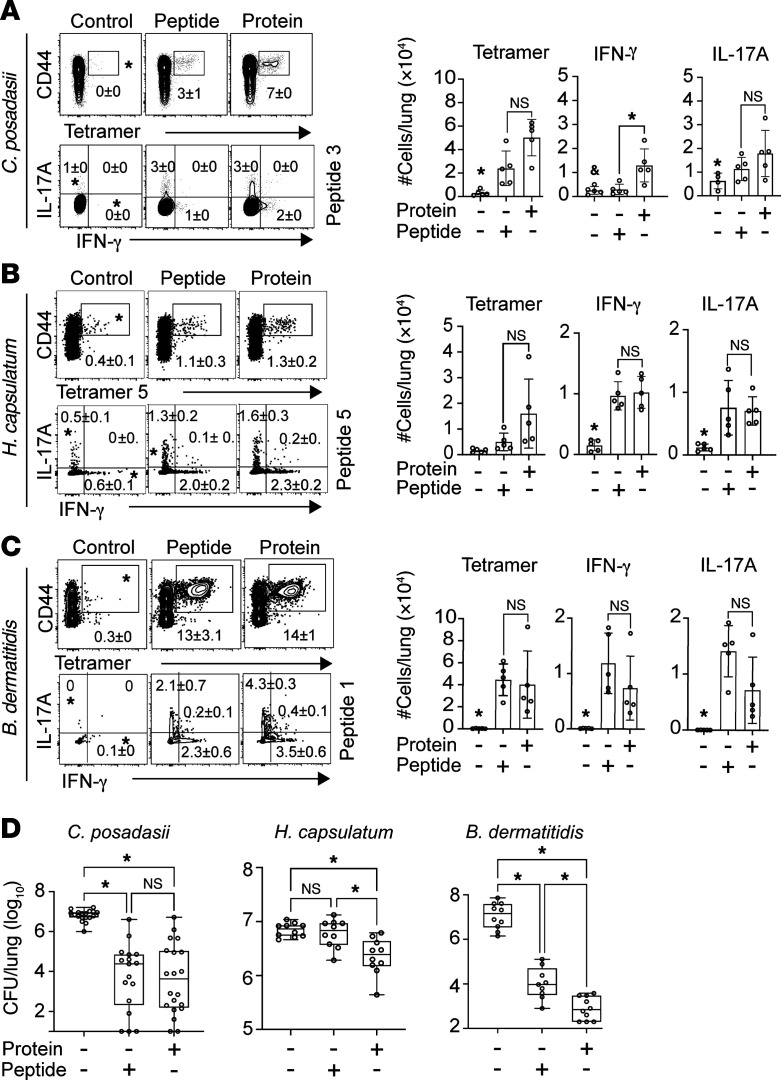
Vaccine protection conferred by immunodominant peptide versus the full-length protein homolog in WT C57BL/6 mice. Mice were vaccinated with *Cp*-Eng2, *Hc*-Eng2, and *Bl*-Eng2 or an equimolar amount of immunodominant peptides from each homolog as described in Methods. Three weeks after the last boost, mice were challenged with a lethal dose of each organism. (**A**–**C**) Lung CD4^+^ T cells were analyzed for the frequency and number of tetramer^+^ cytokine-producing T cells at day 6 (*C*. *posadasii*), day 5 (*H*. *capsulatum*), and day 4 (*B*. *dermatitidis*) after infection (*n* = 5 mice/group). Data are represented as the mean ± SD and were analyzed using 1-way ANOVA with post hoc Dunnett’s test. (**D**) Lung CFU were analyzed 2 weeks after infection when mice in the control group was moribund (*n* = 10 mice/group). CFU are expressed as log_10_ and plotted using box-and-whisker plots with error bars showing minimum and maximum values. *P < 0.05 versus all other groups; ^&^*P* < 0.05 versus protein-vaccinated mice. Data were analyzed using 1-way ANOVA with post hoc analysis by Dunnett’s test.

Vaccination with full-length recombinant proteins offers the advantage that multiple epitopes likely induce a polyclonal and multi-epitope–driven T cell response that could result in optimal protection. Alternatively, some epitopes could activate Tregs that dampen a vaccine benefit (12). Thus, identifying protective T cell epitopes and combining them in multi-epitope vaccines could augment vaccine efficacy (13, 14). Here, we investigated CD4^+^ T cell responses and protective efficacy elicited by immunodominant peptides versus full-length recombinant Eng2 homologs. Vaccination with peptide 3 from *Cp*-Eng2 generally induced the expansion of tetramer^+^ CD4^+^ T cells and the development of Th1 and Th17 cells (Figure 5A). Similarly, peptides P1 and P5 from *Hc*-Eng2 and P1 from *Bl*-Eng2 each induced tetramer^+^ Th1 and Th17 cells that were comparable with the CD4^+^ T cell responses in mice vaccinated with the respective full-length protein (Figure 5, B and C). Although the tetramer response to vaccination with peptide 1 in *Cp*-Eng2 was lower than that for the full-length protein (Figure 5A), peptide 1 reduced lung CFU of *C*. *posadasii* by several logs, similar to that with the full-length protein (Figure 5D). Likewise, vaccination with peptide 3 from *Bl*-Eng2 reduced lung CFU similar to what was observed with the full-length protein. Conversely, vaccination with P1 and P5 from *Hc*-Eng2 combined did not reduce lung CFU (Figure 5D). This was likely due to the overall modest protection achieved by the *Hc*-Eng2 protein. Nevertheless, the peptide vaccine was as effective as the full-length protein vaccine in protecting against *C*. *posadasii* and *B*. *dermatitidis*.

### Protective efficacy of Eng2 homologs and mapping of T cell epitopes in humanized DR4 mice.

**Figure 6:**
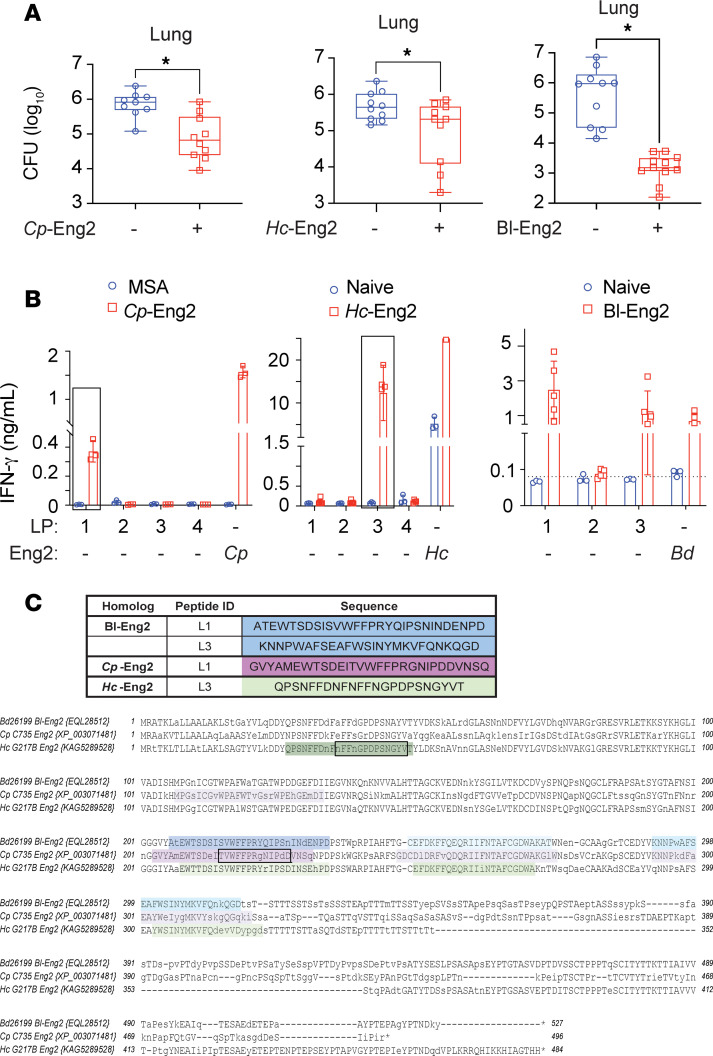
Protective efficacy of Eng2 homologs and mapping of Eng2 epitopes in humanized HLA-DR4 mice. (**A**) Mice were vaccinated with the respective Eng2 homologs and challenged and sacrificed as described in Methods. CFU are expressed as log_10_ and plotted using box-and-whisker plots, with error bars showing minimum and maximum values. (**B**) To assay peptide recognition, splenocytes from mice vaccinated with Eng2 homologs were stimulated ex vivo with the predicted peptides that ranged from 25–30 aa. Cell culture supernatants were assayed for IFN-γ after 5 days of stimulation, and results are presented as the mean ± SD. *P < 0.05 versus the corresponding MSA control groups. (**C**) The sequences of the Eng2 homologs were aligned with the T-coffee algorithm using MacVector 18.5.1. Experimentally determined immunodominant HLA-DR4 epitopes are highlighted in colors. Peptides that elicited the strongest IFN-γ response (*Cp*-P1, *Hc*-P3, and *Bd*-P1) are shaded in darker colors. The 13 mers of the immunodominant epitopes indicated in black boxes are shared between C57BL6 and HLA-DR4 mice.

To translate our findings from C57BL/6 mice to humans, we vaccinated humanized HLA-DR4 (DRB1*0401) transgenic mice that express human MHC II and lack murine endogenous MHC class II molecules (15). Vaccination with *Cp*-Eng2, *Hc*-Eng2, or *Bl*-Eng2 protected HLA-DR4 mice against infection with the corresponding fungus (Figure 6A). HLA-DR4 mice vaccinated with *Cp*-Eng2 also lost less weight and survived longer than did control mice (Supplemental Figure 3, A and B).

To test whether a natural infection induces immunity to Eng2, HLA-DR4 mice were sublethally infected with *H*. *capsulatum*, and their splenocytes were analyzed for cytokine responses to restimulation with *Hc*-Eng2 protein and peptides. CD4^+^ T cells primed during infection produced IFN-γ when restimulated ex vivo with *Hc*-Eng2 protein but not with peptides (Supplemental Figure 3F). Thus, *Hc*-Eng2 was expressed on *H*. *capsulatum* yeast during pulmonary infection, and *Hc*-Eng2–specific CD4^+^ T cells were induced during infection. Below, we demonstrate that PBMCs from individuals that had recovered from *H*. *capsulatum* infection also reacted to *Hc*-Eng2 protein and peptides and therefore harbored antigen-specific memory CD4^+^ T cells.

We next determined the immunogenic peptides of Eng2 proteins that are recognized by human HLA-DR4. Using Immunoinformatics analytics software from Eigenbio, we predicted peptides for each Eng2 homolog. We initially synthesized long peptides (LPs) ranging from 2–30 aa to accommodate recognition of multiple HLAs (in addition to DR4) in testing immunogenicity with the human blood samples. To identify immunogenic peptides, we recalled Eng2 homolog–primed T cells ex vivo with these peptides. Peptides LP1 from *Cp*-Eng2, LP3 from *Hc*-Eng2, and LP1 and LP3 from *Bl*-Eng2 triggered IFN-γ production (Figure 6B). To map the 9 core aa (plus 3 flanking aa on either side) that bind to HLA-DR4 and are recognized by the cognate T cell receptor (TCR), we synthesized 15 mers that overlapped by 14 aa (referred to as peptide walking). GVYAMEWTSDEITVW (peptide 1) from *Cp*-LP1; NFNFFNGPDPSNGYV (peptide 1) from *Hc*-LP3; and PRYQIPSNINDENPD (peptide 15) from *Bd*-LP1; and VKNNPWAFSEAFWSI (peptide 16) from *Bd*-LP3 were identified as the immunogenic 15 mers (Supplemental Figure 3, C, E, and G). The sequence and location of the most immunogenic peptides in each of these Eng2 proteins restricted by HLA class II are depicted in Figure 6C.

To validate our peptide mapping, we created a tetramer to track *Cp*-Eng2–specific CD4^+^ T cells in HLA-DR4 mice. After vaccinating mice with *Cp*-Eng2 and infecting them with *C*. *posadasii*, we detected 4% tetramer^+^ CD4^+^ T cells in the lungs (Supplemental Figure 3D). We assessed vaccine immunity and protection by immunodominant peptides in HLA-DR4 mice. Vaccination induced the generation of IFN-γ–producing CD4^+^ T cells (Supplemental Figure 4, A, C, and E). However, only vaccination with immunogenic peptides from *Cp*-Eng2 was able to reduce lung CFU in HLA-DR4 mice (Supplemental Figure 4, B, D, and F). We used the long immunogenic peptides identified in HLA-DR4 mice to test and confirm reactivity by naive precursor CD4^+^ T cells in humans as outlined below.

### Identification of immunogenic peptides recognized by naive CD4^+^ T cells from humans.

**Figure 7:**
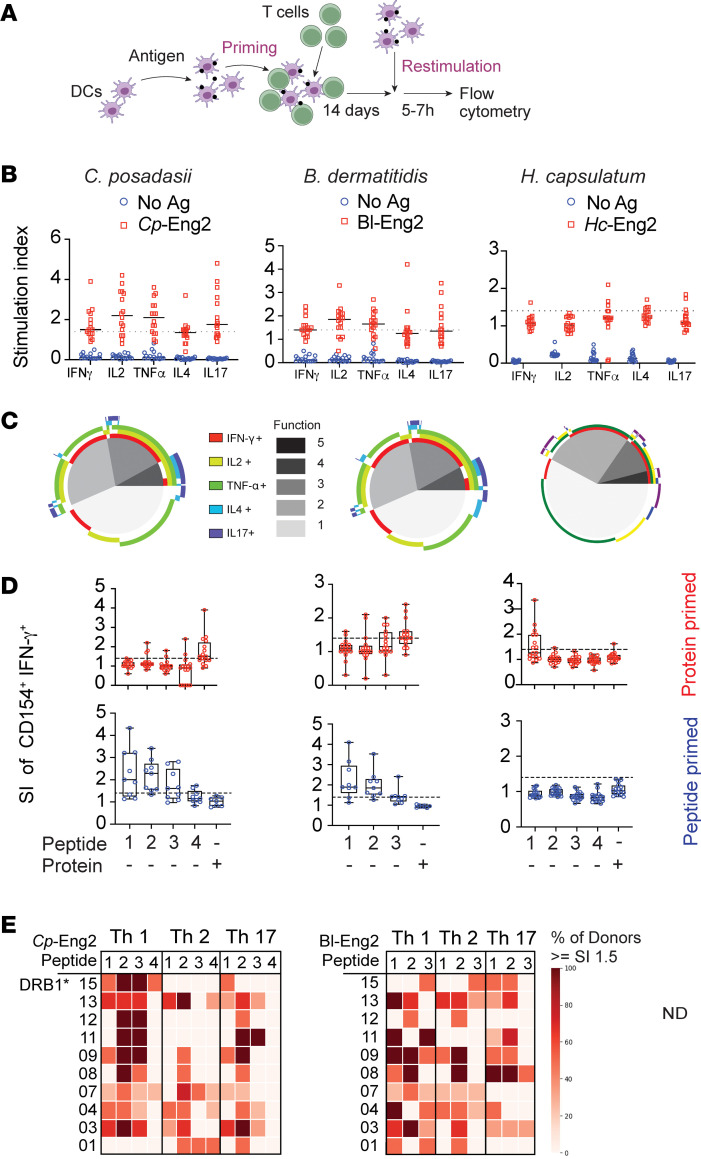
Response of CD4^+^ T cell–naive participants to predicted epitopes. (**A**) Schematic of antigen-priming strategy using Sanofi’s MIMIC System as described in Methods. (**B**) Naive CD4^+^ T cells from 16 healthy donors were primed and restimulated with full length Eng2 protein homologs from *C*. *posadasii*, *B*. *dermatitidis*, and *H*. *capsulatum* using the MIMIC System. Data are expressed as the SI, which is the ratio of cytokines produced by cells that were primed and restimulated versus cultured in medium alone. Ag, antigen. (**C**) Boolean analysis of the multifunctional cytokine response depicted in **B**, illustrating the fraction of activated donor CD4^+^ T cells that produced more than 1 cytokine following Eng2 stimulation (color). “Function” denotes the number of cytokine products. (**D**) Response of donor-derived naive CD4^+^ T cells to priming with protein (top row) or peptide pool (bottom row) and restimulation with individual peptides or protein in the MIMIC System. Data variation is displayed as box-and-whisker plots with variation as noted in Methods. (**E**) Heatmap of responses to peptides P1–P4 with different Th profiles according to individual HLA haplotype among the naive study cohort. Peptide sequences are provided in Supplemental Table 1.

To identify naive human precursor CD4^+^ T cells that recognize Eng2 homologs and their immunogenic peptides, we exploited the Sanofi System as outlined in Methods (Figure 7A). Briefly, antigens were loaded onto CD14^+^ monocytes and naive human CD4^+^ T cells primed for 14 days. Primed T cells were restimulated with “fresh” DCs and stimulated with antigens for 5–7 hours while blocking intracellular protein transport to accumulate cytokines. CD4^+^ T cells from naive donors produced increased intracellular IFN-γ, IL-2, TNF-α, IL-4, and IL-17 after Eng2 antigen priming and restimulation with the antigen compared with medium alone (Figure 7B). The majority of primed CD4^+^ T cells were polyfunctional and produced multiple cytokines, as indicated by Boolean analysis (Figure 7C). The limited responses to *Hc*-Eng2 and peptides likely stemmed from the low precursor cell frequency (Supplemental Figure 2C). Interestingly, peptide-primed CD4^+^ T cells showed higher stimulation indices for activated (CD154^+^) and IFN-γ–producing cells than did the full-length protein–primed cells when restimulated with peptides (Figure 7D). We assessed whether different immunodominant peptides from an individual Eng2 protein influenced the Th phenotypes in an HLA haplotype–specific manner. Peptides 2 and 3 from *Cp*-Eng2 and peptide 1 from *Bl*-Eng2 favored the development of Th1 cells when presented by most HLA-DR haplotypes (Figure 7E), although the sample size was insufficient to achieve statistical significance.

### Memory T cell responses from patients recovered from infection with dimorphic fungi.

**Figure 8:**
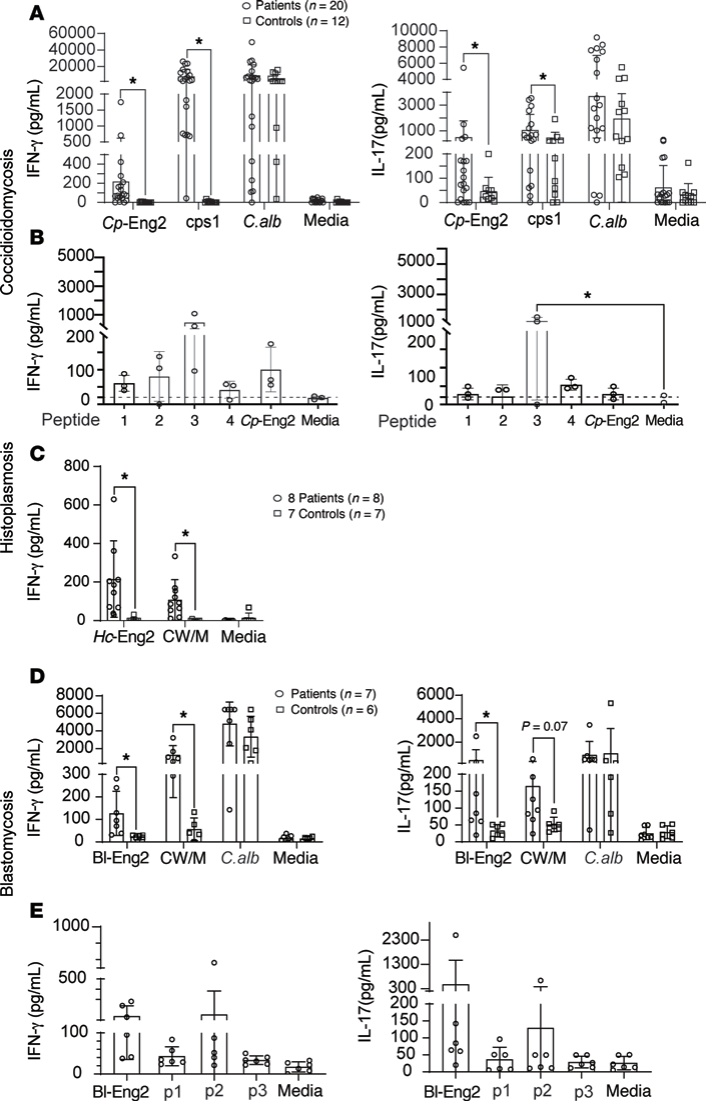
Eng2-specific memory T cells in fungi-exposed patients. Recall response of CD4^+^ T cells from patients with coccidioidomycosis and healthy control individuals after stimulation with (**A**) *Cp*-Eng2 protein or (**B**) peptides. CD4^+^ T cells were expanded by coculturing them with CD14^+^ monocytes in the presence of various stimuli for 7 days. IFN-γ and IL-17 were measured in the culture supernatant by ELISA. Heat-killed *Dcps1* spores were used to confirm the patient had immunity to *C*. *posadasii*, and *Candida* yeast served as a control to exclude anergy. Stimulation media alone served as a negative control. (**C**) Ex vivo response of PBMCs from healthy blood donors previously exposed to *H*. *capsulatum* following 5 days of stimulation with *Hc*-Eng2 or *H*. *capsulatum* CW/M antigen as an indicator of prior infection. Patients with blastomycosis and healthy control individuals were stimulated with Bl-Eng2 protein or positive control antigens (**D**), or Bl-Eng2 peptides (**E**). Each symbol represents a patient or control. Data are presented as the mean ± SD. *P ≤ 0.05, by 2-tailed Mann-Whitney *U* test or 1-way ANOVA with post hoc analysis by Dunnett’s test. Peptide sequences are provided in Supplemental Table 1.

We studied a total of 35 individuals who recovered from infection with *C*. *posadasii*, *H*. *capsulatum*, or *B*. *dermatitidis* infection and 25 healthy control individuals. We assessed whether patients harbored a pool of memory CD4^+^ T cells specific for the corresponding Eng2 protein homolog and respective immunogenic peptides identified above. PBMCs from 20 patients recovered from confirmed *C*. *posadasii* infection all responded to positive control antigens (attenuated *C*. *posadasii* strain *DCps1* [refs. 16–19] and *C*. *albicans*) and 16 (80%) produced IFN-γ or IL-17 in response to stimulation with *Cp*-Eng2 protein (Figure 8A). Twelve control individuals did not respond to *Cp*-Eng2, and all but 1 responded to a positive control. Five of the patients manifested disseminated disease at the time of diagnosis (4 with meningitis). Each was receiving antifungal treatment at the time of testing — several for a number of years. Individuals with prior disseminated *C*. *posadasii* infection produced IFN-γ or IL-17 in response to *Cp*-Eng2 when tested. Seven patients who had recovered from *C*. *posadasii* were also assessed for responses to *Cp*-Eng2 peptides 1–3 (Figure 8B), and peptide 3 was found to elicit the strongest response (Figure 8B).

Approximately 80% of people who live in the *H*. *capsulatum–*endemic region of Ohio have evidence of prior infection with the fungus. We identified individuals previously infected with *H*. *capsulatum* by testing their PBMCs for production of IFN-γ in response to cell wall membrane (CW/M) extract from *H*. *capsulatum* as described previously (20). Eight of the 10 individuals tested had evidence of CW/M reactivity, indicating prior infection; all 8 also responded strongly to *Hc*-Eng2 (Figure 8C), often more so than to CW/M. Seven healthy controls participants from outside the endemic area failed to respond to *Hc*-Eng2 (and CW/M), confirming the specificity of the response to *Hc*-Eng2.

Six of the 7 patients who had recovered from confirmed infection with *B*. *dermatitidis* and responded to the positive control stimulus of CW/M extract from *B*. *dermatitidis* also responded to *Bl*-Eng2 by producing IFN-γ or IL-17 (Figure 8D). Two other individuals with a confirmed case of acute infection in whom treatment had just been initiated failed to respond to a positive control stimulus and were not evaluable. Six healthy control participants did not respond to *Bl*-Eng2. Cells from 6 patients were also tested for responses to *Bl*-Eng2 peptides (Figure 8E). Peptide 2 elicited the strongest IFN-γ and IL-17 response.

These data suggest that most individuals who had recovered from infections with these endemic mycoses responded to the corresponding Eng2 homolog. A number of these individuals also responded to immunogenic peptides identified in our studies of humanized mice and infection-naive individuals.

We tested 15 patients who had recovered from infection for cross-reactivity to the 3 Eng2 proteins: 7 patients with *C*. *posadasii* and 4 patients each with *H*. *capsulatum* or *B*. *dermatitidis*. Each person had responded to the Eng2 protein corresponding to their infection. Of the 7 patients with *C*. *posadasii* infection, 4 had cross-reactions to *Hc*-Eng2 but not to *Bl*-Eng2. Of the 4 individuals with *H*. *capsulatum* infection, none had a cross-reaction to the other Eng2 proteins. Of the 4 patients tested for *B*. *dermatitidis*, each cross-reacted to both *Hc*- and *Cp*-Eng2. Thus, among 15 individuals tested for cross-reactivity, 8 cross-reacted to 1 of the heterologous Eng2 proteins. This finding suggests that there are shared CD4^+^ T cell epitopes within the Eng2 proteins that elicit responses to heterologous Eng2 among patients with an endemic mycosis, although these epitopes are distinct from those eliciting protection in animal models.

## Discussion

Endemic systemic mycoses are invasive fungal diseases that usually occur in previously healthy individuals and are not limited to hosts with impaired cellular or humoral immunity. We report here that Eng2 from the cell wall of the causative fungi elicited immunity in both humans and mice and protected the latter against experimental lethal pulmonary infection. Thus, vaccination with the respective Eng2 homolog protected C57BL6 and humanized HLA-DR4 mice against corresponding infections with *C*. *posadasii*, *H*. *capsulatum*, or *B*. *dermatitidis*. Despite some sequence similarity among the Eng2 homologs, we did not observe cross-protection by *Bl*-Eng2 against infection with *C*. *posadasii* or *H*. *capsulatum*. Nevertheless, each of the respective homologs induced resistance in mice and adaptive immunity in humans during their infections. Thus, persons recovered from infection displayed reactivity to the respective protein, suggesting that these antigens could have diagnostic value for the detection of infection.

By mapping immunodominant T cell epitopes for each Eng2 homolog, we learned that the peptides differed in sequence and location. This contrasts with our findings with the conserved antigen, calnexin, that confers vaccine protection against *C*. *posadasii*, *H*. *capsulatum*, and *B*. *dermatitidis* by an identical 13 aa epitope (5). Corresponding 1807 TCR transgenic (calnexin-specific) T cells expanded not only after infection with *B*. *dermatitidis* but also after challenge with other dimorphic fungi including *C*. *posadasii* and *H*. *capsulatum* (21). Furthermore, infection with other clinically relevant ascomycetes such as *Aspergillus fumigatus*, *Fonsecaea pedrosoi*, and *Pseudogymnoascus destructans* triggered the expansion of calnexin peptide–MHC II tetramers (5). The calnexin study thus demonstrated the possibility of exploiting a single conserved epitope/antigen as a target for the development of broadly reactive antifungal vaccines. However, a limitation for the development of a calnexin-specific antifungal vaccine is the low precursor frequency of naive endogenous CD4^+^ T cells and the modest expansion and protective efficacy after vaccination and infection (5). We overcame this limitation here with the discovery of Eng2 homologs. The precursor frequency of the immunodominant epitopes of *Cp*- and *Bl*-Eng2–specific naive endogenous CD4^+^ T cells in C57BL/6 mice was 97–130 cells, compared with a frequency of only 29 cells for calnexin-specific precursor cells (5) and 32–47 for *Hc*-Eng2–specific precursor cells. Consequently, vaccine-induced expansion of antigen-specific T cells and protection were much higher for experimental *C*. *posadasii* and *B*. *dermatitidis* infection compared with the expansion and protection seen with *Hc*-Eng2. Our data support the concept that a large population of T cell precursors is associated with increased TCR diversity and a greater primary immune response and explain why immune responses to some peptides are stronger than others (22).

We chose C57BL/6 mice as the strain to model these infections given its common use. This strain harbors a common structural defect in dectin 1 associated with a splice variant known as dectin 1B (23). Dectin 1B thus lacks a stalk region present in the full-length dectin 1A isoform. The shorter variant arises from the deletion of exon 3 during splicing of *CLEC7A*, which encodes dectin-1. The defect is associated with susceptibility to coccidioidomycosis in C57BL/6 mice (24). Polymorphisms in dectin 1 have also been linked to susceptibility to disseminated coccidioidomycosis in patients (25). We chose this strain of mouse despite its susceptibility to this mycosis as a means of rigorously testing the efficacy of Eng2-mediated protection in a manner that would be relevant for human populations at risk. Our findings imply that even vulnerable individuals with genetic predisposition(s) can acquire vaccine immunity.

We translated our findings from C57BL/6 mice to humans. We first vaccinated humanized HLA-DR4 mice to confirm protection and identify the immunogenic T cell epitopes. Vaccination with all three Eng2 homologs protected HLA-DR4 mice against infection with the respective dimorphic fungi, suggesting that human T cells could be primed during immunization. For example, *Bl*-Eng2 protects against *B*. *dermatitidis*, and *Cp*-Eng2 protects against C. *posadasii*, and *Hc*-Eng2 protects against *H*. *capsulatum*. This idea was supported by the observation that vaccination with Eng2 homologs and peptides primed HLA-DR4–restricted CD4^+^ T cells that produced IFN-γ upon recall in vitro. Since the immunogenic epitopes for the three Eng2 homologs differed in aa sequence and/or location, it is conceivable that they could be engineered as a multi-epitope vaccine that would be broadly reactive and elicit protective immunity against endemic systemic mycoses. A recombinant chimeric polypeptide antigen (rCpa1) has been genetically engineered, encompassing 5 epitopes derived from *C*. *posadasii–*specific aspartyl protease (Pep10), a mannosidase (Amn1), and phospholipase B (Plb) antigens (14, 26). The vaccine elicits a mixed Th1 and Th17 immune response and confers protection in C57BL6 and HLA-DR4 mice against multiple isolates of *C*. *posadasii* and *Coccidioides* *immitis* (27).

We used long “promiscuous” peptides identified in HLA-DR4 mice to survey infection-naive individuals for reactive T cell precursors. CD14^+^ monocytes from these individuals, loaded with Eng2 homologs or the immunogenic peptides, primed and expanded autologous naive CD4^+^ T cells and triggered the production of multiple cytokines. Interestingly, peptide-primed CD4^+^ T cells produced larger amounts of cytokines when recalled with the corresponding peptides than did protein-primed T cells. The increase in cytokine production likely occurred because the peptide-primed T cells were not subject to clonal competition, whereas protein-primed T cells had to compete for antigen and deal with clonal competition. While only peptide 1 for *Cp*-Eng2 was immunogenic in vaccinated HLA-DR4 mice, peptides 1–3 were found to be immunogenic in the Modular Immune In vitro Construct (MIMIC) System assay using naive T cells. The diversity of HLA haplotypes of the human T cells likely accounted for the recognition and immunogenicity of the additional peptides.

We hypothesized that human infection with these dimorphic fungi elicits adaptive immunity to the Eng2 homologs, given their immunodominance and vaccine effect. Our observation that pulmonary infection of HLA-DR4 mice with *H*. *capsulatum* yeast primed antigen-specific CD4^+^ T cells that produced IFN-γ upon recall in vitro with *Hc*-Eng2 supported this hypothesis and prompted us to study Eng2-specific T cell responses in individuals recovered from the corresponding infections. Such individuals harbored memory T cells that produced cytokines in response to recall in vitro with Eng2 homologs, indicating that human T cells recognized this antigen during natural infection. These memory T cells were selected by HLA restriction and subjected to antigen and clonal competition. Peptides 1–3 from *Cp*-Eng2 triggered IFN-γ production by memory T cells, but the epitope hierarchy was dominated by peptide 3, which was different than the balanced epitope hierarchy we observed with naive T cells in the MIMIC System. Thus, cytokine production by human memory T cells in response to immunogenic peptides is likely a consequence of several factors, including HLA restriction (e.g., some peptides are not presented by each HLA during infection), immunodominance, precursor frequency and hierarchy of T cell epitopes in response to the fungal infection, and the polyclonal nature of the T cell response.

The findings here could lead to the generation of a vaccine against all 3 fungi by reverse-engineering the endoglucanase from 1 strain to express the other T cell epitopes with site-directed mutagenesis. Alternatively, one could imagine developing mRNA or antigen cocktails in a single vaccine against these 3 fungal species. Furthermore, since patients with prior infection demonstrated reactivity to 1 or more Eng2 proteins, individuals with recent or remote infection could be identified and removed from the target population for vaccination, as reinfection is uncommon, particularly for coccidioidomycosis and blastomycosis.

A limitation of this study is the small number of patients tested for endemic mycoses, with even smaller numbers when one considers each individual mycosis. Larger numbers of patients should be studied to confirm and extend these findings.

Among patients, some cross-reactivity to heterologous Eng2 proteins is not surprising, since Eng2 is shared among these 3 fungi. Thus, while the immunodominant epitopes may be distinct between the Eng2 proteins, there are likely to be minor, subdominant epitopes that are shared. Surface galactomannan on the endemic fungi presents an analogous situation, as it is conserved, while showing some structural differences. Thus, clinicians often find that the urine antigen test is positive for both *Histoplasma* and *Blastomyces* in patients with blastomycosis, as an example. The result is of value in establishing the presence of a mycotic infection and allows the clinician to have a high level of confidence that a given patient is infected with one of these fungi. The clinical and epidemiological features help to determine the likely infection.

In summary, we conclude from this study that Eng2 homologs were expressed on the infectious or tissue form of these dimorphic fungi during natural infection and elicited functional memory CD4^+^ T cells with clinical significance. Our findings have implications for strategies to prevent and also reliably diagnose the systemic endemic mycoses of North America, which represent a substantial and growing public health problem.

## Methods

### Sex as a biological variable.

Both male and female C57BL/6 and HLA-DR4 mice were used in the vaccination experiments, and similar outcomes were found. Our human studies included both male and female participants without explicit analysis dedicated to determination of sex as a biological variable.

### Fungi.

WT *B*. *dermatitidis* strain ATCC 26199, *C*. *posadasii* virulent clinical isolate C735 (American Type Culture Collection [ATCC] 96335), and *H*. *ccapsulatum* G217B and *C*. *albicans* strain ATCC SC5314 were used for this study. *B*. *dermatitidis* 26199 was grown as yeast on Middlebrook 7H10 agar with oleic acid–albumin complex (MilliporeSigma) at 39°C. Saprobic phase *C*. *posadasii* was grown on 2× GYE agar (2% glucose, 1% yeast extract, 1.5% agar) at 30°C to produce spores as reported previously (28). Culturing and harvesting of *C*. *posadasii* were done in a biosafety level 3 (BSL3) laboratory located at the UW-Madison. *H*. *capsulatum* was grown on brain heart infusion agar blood at 37°C for 15 days. *Candida* yeast was grown on yeast extract, peptone, and dextrose agar at 30°C.

### Mouse strains.

Male and female mice were 7 to 8 weeks of age at the time of these experiments. Inbred WT C57BL/6 mice (stock no. 664) obtained from The Jackson Laboratory were bred at our facility. A breeding colony of HLA-DR4 (DRB1*0401) transgenic mice that express human MHC class II molecules (15) (Taconic, model no. 4149) was used in this study. HLA-DR4 mice were engineered from a C57BL/6 background and backcrossed with MHC class II–deficient mice lacking IA and IE alleles to eliminate production of endogenous murine MHC class II molecules. Mice were housed and cared for in a specific pathogen–free environment in our animal facility, as per guidelines of the UW-Madison Animal Care Committee, who approved all aspects of this work. Mice were transported to the animal BSL3 (ABSL3) laboratory for challenge with live *C*. *posadasii* spores.

### Immunoinformatics and epitope mapping.

Immunogenic peptides were predicted using the Eigenbio immunoinformatics platform described elsewhere (29, 30). Briefly, the mean and SD of the natural log of IC_50_ MHC II allele binding for each sequential 15 aa peptide in the protein were predicted by artificial neural network ensembles using algorithms based on vectors derived from the principal components of the physical and chemical characteristics of each aa. Mean predicted binding was standardized to a zero mean unit variance (normal) distribution within the protein to provide a relative competitive index of predicted binding for each peptide in the protein. This places binding predictions of all MHC alleles on the same scale. This metric is expressed in SD units relative to the mean for that protein. Comparison with other prediction systems indicates that a predicted binding affinity of less than –1 SD unit below the mean is a probable epitope (31). Predicted peptides were synthesized at greater than 75% purity by GenScript and provided as lyophilized material in measured amounts of peptide. Each peptide was analyzed by GenScript for purity using high-performance liquid chromatography (HPLC), mass spectrometry, and nitrogen analysis. Depending on their solubility, peptides were dissolved in water or 100% DMSO. Stock solutions of each peptide were adjusted to 10 mM and stored at –80°C.

### Generation and purification of recombinant Eng2 homologs.

*Cp*-Eng2 and *Hc*-Eng2 were cloned and expressed in *P*. *pastoris* and *E*. *coli* using standard recombinant techniques and have been described for *Bl*-Eng2 (32). Recombinant proteins were purified using Ni-NTA agarose (Qiagen) according to the manufacturer’s protocol and dialyzed against PBS. The purity of recombinant proteins was assessed by SDS-PAGE and silver staining.

We tested recombinant Eng2 enzymatic activity as a measure of quality control. We investigated cleavage of b-1-3 glucan because the conserved endoglucanase domains closely resemble *Laminarinase Lam16A* from *Phanerochaete chrysosporium* (33). We documented Eng2 enzymatic activity using the dinitrosalicylic acid (DNS) method (34) (Supplemental Figure 5). The DNS method is used to estimate the concentration of reducing sugars in enzymatic hydrolysates. Briefly, the DNS reagent is mixed with a sample and heated to catalyze the reaction, and the visible absorbance of the products (particularly 3-amino-5-nitrosalicylic acid) is measured as described previously. We added 20 μL of 0.75% laminarin (substrate) (MilliporeSigma) to 200 μg enzyme (laminarinase, positive control) (MilliporeSigma) or Eng2. After plates were incubated at 50°C for 10 minutes, 100 μL DNS reagent was added and incubated at 95°C for 5 minutes. After cooling, plates were read at 540 nm. Absorbance was converted to micromolars of reduced sugar according to a standard glucose curve.

### Generation of rabbit anti-Eng2 antibody and surface staining of dimorphic fungi.

Rabbit polyclonal antibody was generated by Envigo Research and Model Service. Briefly, rabbits were vaccinated 3 times with 1 mg recombinant vaccine of each Eng2 homolog, and serum was harvested on day 112 after the first vaccination.

Yeast or spherules were washed and fixed in 4% formaldehyde for 15 minutes at room temperature, washed, and then resuspended in blocking buffer containing 1% BSA in PBS plus 0.1% Tween 20 for 15 minutes. After washing, fungi were stained with the corresponding polyclonal anti-Eng2 rabbit antibodies at a 1:800 dilution and incubated at 4°C overnight. After washing, fungi were stained with 5 μg/mL secondary donkey anti– rabbit IgG antibody conjugated with Alexa Fluor 647 (Invitrogen, Thermo Fisher Scientific, catalog A-31573) for 2 hours. After washing, fungi were stained with 10 μg/mL Calcofluor White Blue (MilliporeSigma) for 20 minutes and washed again. Samples were resuspended in mounting medium (VECTASHIELD Vibrance Antifade Mounting Medium, Vector Laboratories), transferred onto microscope slides, covered with a coverslip (Circular Coverglass 12 mm, Electron Microscopy Sciences), and stored at 4°C. A Nikon A1R confocal microscope was used to capture the slide images.

### Vaccination and fungal infection.

Ten micrograms of *Bl*-Eng2, 20 mg *Cp*-Eng2, or 30 mg *Hc*-Eng2 or equimolar amounts of synthetic peptides were loaded into GCPs. For vaccination with peptide pools, individual peptides were resuspended in DMSO, and equal amounts of each peptide were pooled and mixed with 50% CAF01 (Statens Serum Institute) or 10% adjuplex (Empirion). Mice were vaccinated s.c. with Eng2 full-length proteins 3 times, 2 weeks apart as described before (35), with antigen loaded into GCPs; controls in these experiments were GCPs loaded with mouse serum albumin (MSA) (Gary Ostroff, University of Massachusetts Chan Medical School). In limited experiments, Freund’s adjuvant (MilliporeSigma) was used for vaccination. Two weeks after the vaccine boost, mice were challenged intratracheally (i.t.) with 2 × 10^4^ WT *B*. *dermatitidis* yeast, 100–150 *C*. *posadasii* spores, or 10^6^ to 10^7^ *H*. *capsulatum* yeast in 30 mL PBS. At post-infection days 4–6, lung T cell responses were analyzed, and lung CFU were counted. Two weeks after infection, when control or unvaccinated mice were moribund, fungal burden was determined by plating lung CFU.

### Generation of MHC II tetramers.

Tetramers for detection of Eng2-specific CD4^+^ T cells in C57BL6 were generated at the NIH Tetramer Core facility at Emory University Atlanta, Georgia, USA). A tetramer that recognizes *Cp*-Eng2–specific CD4^+^ T cells in HLA-DR4 mice was generated by ProImmune (Oxford, England).

### Human donors and PBMC isolation.

For MIMIC assays, PBMCs were obtained from healthy donors who enrolled in a Sanofi VaxDesign apheresis study (protocol CRRI 0906009) after providing informed consent. Leukocytes were enriched by centrifugation over a Ficoll-Histopaque PLUS (General Electric Healthcare) density gradient (33), collected at the gradient interface, washed, and cryopreserved in IMDM (Lonza) containing autologous serum and 10% DMSO (MilliporeSigma). Blood products were negative for blood-borne pathogens as detected by standard assays. For blood samples from patients recovered from infection, cases of blastomycosis and coccidioidomycosis were confirmed by culture or histopathology. For histoplasmosis, we received deidentified blood from the Hoxworth Blood Bank of the University of Cincinnati College of Medicine (Cincinnai, Ohio, USA).

### MIMIC System CD4^+^ T cell LTE assay.

Sanofi’s proprietary MIMIC System was used to assay whether naive human CD4^+^ T cells recognize peptides in Eng2 homologs. Human donors were selected on the basis of their HLA-DRB1 haplotypes to achieve a diverse sampling of more commonly expressed HLA-types. CD14^+^ cells and CD4^+^ T cells from donor PBMCs were purified using CD14-positive and CD4-negative selection isolation kits (STEMCELL Technologies). CD14^+^ cells were treated with recombinant IL-4 and GM-CSF (R&D Systems) to promote DC differentiation. One week after isolation, these cytokine-derived DCs (CDDCs) were loaded with antigens at doses ranging from 1–5 mg/mL. A vehicle control was included to assess donor background responses. Antigen-loaded DCs were cocultured with purified donor-matched CD4^+^ T cells for 14 days as part of the primary stimulation phase. Unpulsed DCs, or those pulsed with irrelevant antigen, served as negative controls.

After 2 weeks, cocultured cells were harvested and restimulated with fresh CDDC loaded with the same protein or peptide or related antigen. Cells were then assayed for CD154^+^ expression (activation) (anti–CD154-APC-Cy7) (BioLegend: clone 24-31, catalog 310822) and intracellular cytokines. To assay intracellular cytokines, cultures received brefeldin A for 5–7 hours of restimulation. After restimulation, cells were fixed, permeabilized, and stained with a panel of antibodies to detect intracellular IFN-γ (anti–IFN-γ–PECy7, eBioscience: clone 4S.B3, catalog AB2535390), TNF-α (anti–TNF-PE, BioLegend: clone MAB11, catalog 502909), and IL-2 (anti–IL-2–APC (BioLegend: clone W19046A, catalog 310605) (demark Th1 cells), IL-4 (anti–IL-4–PerCP-Cy5.5, BioLegend: clone MP4-25D2, catalog 500822) (Th2 cells) and IL-17 (anti–IL-17 A488, BioLegend: clone BL168, catalog 512307) (Th17 cells). Data were analyzed with FlowJo, GraphPad Prism, Microsoft Excel, and Spice (version 5.3). The stimulation indices (SIs) were calculated as a ratio of the frequency of T cells producing cytokines in response to peptide or protein compared with unstimulated T cells.

### ELISA.

Cytokine concentrations in cell culture supernatants were determined using IFN-γ and IL-17A Duoset ELISA kits (R&D Systems) according to the manufacturer’s instructions.

### Tetramer pulldown.

Miltenyi LS columns on a quadroMACS magnet were used to enrich tetramer^+^ cells from secondary lymphoid organs (SLOs). Spleens and draining lymph nodes were harvested and mashed through 40 μm filters. RBCs were lysed with ACK buffer (Thermo Fisher Scientific). Samples were washed with RPMI and resuspended in cold sorter buffer (PBS with 2% FBS) to a volume twice the size of the pellet. Fc block (2 μL) was added to each sample and incubated for 5 minutes before adding tetramer (5–25 nM). Tetramer staining was done for 1 hour at room temperature in the dark. Samples were then washed and kept on ice. Miltenyi anti-PE microbeads (100 μL) (Miltenyi 130–048–801) were added to each sample and incubated for 30 minutes on ice, and then washed and resuspended in 3 mL sorter buffer. LS columns were pre-wet and samples filtered through 40 μm filters into fresh columns. Columns were washed with cold sorter buffer thrice before eluting bound fractions. Fractions were stained with Invitrogen’s LIVE/ DEAD stain and surface markers. AccuCheck Counting Beads (50 mL) (Invitrogen, Thermo Fisher Scientific, PCB100) were added to each sample to determine the total number of tetramer^+^ cells.

### T cell stimulation and flow cytometry.

Lungs were dissociated in Miltenyi MACS tubes and digested with collagenase (1 mg/mL) (MilliporeSigma) and DNase (1 μg/mL) (MilliporeSigma) for 25 minutes at 37°C. Digested lungs were resuspended in 5 mL of 40% Percoll, and 3 mL of 66% Percoll was underlaid (GE Healthcare, catalog 17–0891–01). Samples were spun for 20 minutes at 2,000 rpm at room temperature. Lymphocytes in the buffy coat were harvested and resuspended in RPMI (10% FBS, 1% penicillin and streptomycin). For T cell stimulation ex vivo, cells were incubated at 37°C for 5 hours with 5 μM peptide and 1 μg anti–mouse CD28 (BD, catalog 553294). After 1 hour, BD GolgiStop (BD, catalog 554724) was added to samples. FACS samples were stained with Invitrogen’s LIVE/DEAD stain and Fc block for 10 minutes at room temperature. Cells were stained with tetramer for 1 hour at room temperature, or for surface antigens (anti–CD45-AF488, BioLegend: clone 30-F11, catalog 103122); anti–PerCpCy5.5 (BioLegend: clone 53.67, catalog 100734); anti–CD90.2-BV421 (BioLegend: clone 30H12, catalog 105341); anti–CD44-BV786 (BioLegend: clone IM7, catalog 103059); anti–CD4-BUV737 (BD Bioscience: clone RM4-5, catalog 612844) and dump channel antibodies (anti–CD11c-APC (BioLegend: clone N418, catalog117310); anti–CD11b-APC (BioLegend: clone M1/70, catalog 101212); anti–NK1.1-APC (BioLegend: clone P136, catalog 108710); anti–B220-APC (BioLegend: clone RA3-6B2, catalog 103212) or intracellular targets (anti–IFN-γ–PE-Cy7 (BioLegend: clone XMG1.2, catalog 505826); anti–IL-17A–PE (BioLegend: clone TC11-18H10.1, catalog 506904); anti–IL-5–BV421 (BioLegend: clone TRFK5, catalog 504311); anti–IL-13–eFlour450 (eBioscience: eBio13A, catalog 48-7133-82) for 20 minutes at 4°C. Transcription factors were stained on primed, resting cells using the Foxp3 Transcription Factor Staining Kit (eBioscience, catalog 00–5523–00). All panels included a dump channel (Dump markers: CD11b, CD11c, NK1.1, CD19 B220). AccuCheck Counting Beads (50 mL) (Invitrogen, Thermo Fisher Scientific, PCB100) were added to samples to determine absolute cell counts. Samples were acquired on a LSR Fortessa (BD Biosciences).

### Statistics.

A 2-tailed, Mann-Whitney U test was primarily used to analyze the differences between 2 treatment groups for cytokine ELISAs, cytokine concentrations, calculations of the number of lung- infiltrating immune cells, and percentages of specific cytokine-producing T cells. In some instances, 1-way ANOVA was used when comparing multiple groups, and when a result was significant, a Tukey’s or Dunnett’s post hoc test was used to adjust for multiple comparisons. Differences in fungal burden (expressed as CFU) between 2 groups were analyzed using the Mann-Whitney *U* test for ranking data. For comparison of fungal burden among 3 or more groups of mice, the Kruskal-Wallis test, a nonparametric ranking method was used. Survival data were examined by the Kaplan-Meier test using log-rank analysis to compare survival plots as reported previously (6). A *P* value of 0.05 or less was considered statistically significant. Comparisons in many experiments yielded *P* values at or below the value of 0.05, however we consistently used only 1 asterisk throughout to denote any statistically significant difference, regardless of the exact *P* value below 0.05.

Data in the figures are illustrated in either of 2 ways: arithmetic mean and SD indicating the typical amount of variation of the dataset. Alternatively, in some figures, we depicted variation with box-and-whisker plots showing the median and minimum and maximum values for all the data. The box represents the middle 50% of the data, extending from the first to the third quartile; the line in the box represents the median.

### Study approvals.

Animal studies adhered to protocol M005891 approved by the IACUC of UW-Madison. The studies were compliant with provisions established by the Animal Welfare Act and the Public Health Services Policy on the Humane Care and Use of Laboratory Animals. Studies involving the collection of blood samples from study participants were approved by Human Subject Institutional Review Boards at the UW-Madison, the University of Cincinnati, and the UCD. Written informed consent was obtained from study participants prior to their participation. Mice were housed and cared for in a specific pathogen–free environment in our animal facility as per guidelines of the University of Wisconsin Animal Care Committee, who approved all aspects of this work.

### Data availability.

Values for all data points in graphs are reported in the Supporting Data Values file.

## Author contributions

MW, BSK, GCK, and CYH designed the research studies. UJO, CLT, LDSD, HD, AC, and GSD performed experiments. EJH performed peptide analysis and predictions. UJO, CLT, LDSD, HD, AC, and GSD acquired data. UJO, CLT, LSD, HD, and AC analyzed data. GRT and GSD provided reagents and samples. UJO and CLT wrote the manuscript. MW and BK edited the manuscript.

## Funding support

This work is the result of NIH funding, in whole or in part, and is subject to the NIH Public Access Policy. Through acceptance of this federal funding, the NIH has been given a right to make the work publicly available in PubMed Central.

- NIH grants: BAA-NIAID-DAIT-AI201800007, contract no. 75N93019C00064, R01AI93553 (to MW); R01AI040996 (to BK and MW); R01 AI168370 (to BK); U01 AI124299 (to BK); R37 AI035681 (to BK); R01AI178010 (to GSD); R01AI135005 (to CYH); and U19AI166761 (to CYH).
- Division of Intramural Research, NIAID, NIH (to LSD).
- American Heart Association Postdoctoral Fellowship no. 835129 (to LSD).
- NIH shared instrumentation grant 1S100OD018202-01 and University of Wisconsin Carbone Cancer Center Support grant P30 CA014520 (funding of BD LSR Fortessa for flow sample processing at the University of Wisconsin Carbone Cancer Center [UWCCC] Flow Core Facility).
- UW-Madison Department of Biochemistry Endowment (support for microscopy performed at the UW-Madison Biochemistry Optical Core, RRID:SCR_023952).

## Supplementary Material

Supplemental data

Supporting data values

## Version 1 10/08/2025

In-Press Preview

## Version 2 11/17/2025

Electronic publication
