# Supplementary material for: Endoglucanase 2 (Eng2), a shared immunodominant antigen in dimorphic fungi that elicits immunity during infection
Source: J Clin Invest. 2025 Sep 25;135(22):e191103. doi: 10.1172/JCI191103 (PMC12618052; doi:10.1172/JCI191103)
Supplement: Supplemental data [file jci-135-191103-s026.pdf]

SUPPLEMENTAL INFORMATION

Supplemental Figure 1 (related to figure 1)

Supplemental Figure 2 (related to figure 3)

Supplemental Figure 3 (related to figure 6)

Supplemental Figure 4 (related to figure 6)

Supplemental Figure 5 (related to Methods) Supplemental Table 1. Peptides used to stimulate cells in healthy donors and patients with infection (related to main Figures 7 and 8)

Supplementary figure 1 related to figure 1

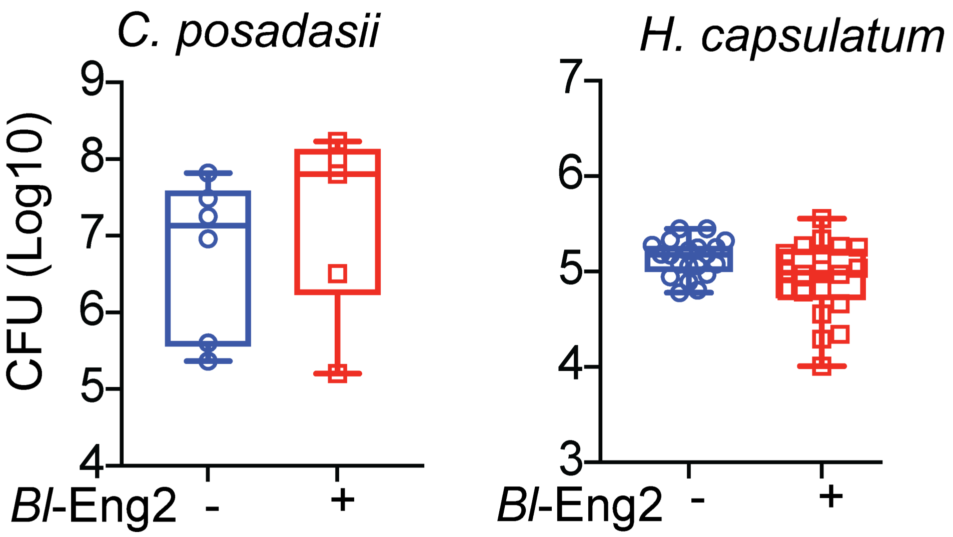

Supplementary Fig 2 related to Figure 3

A.

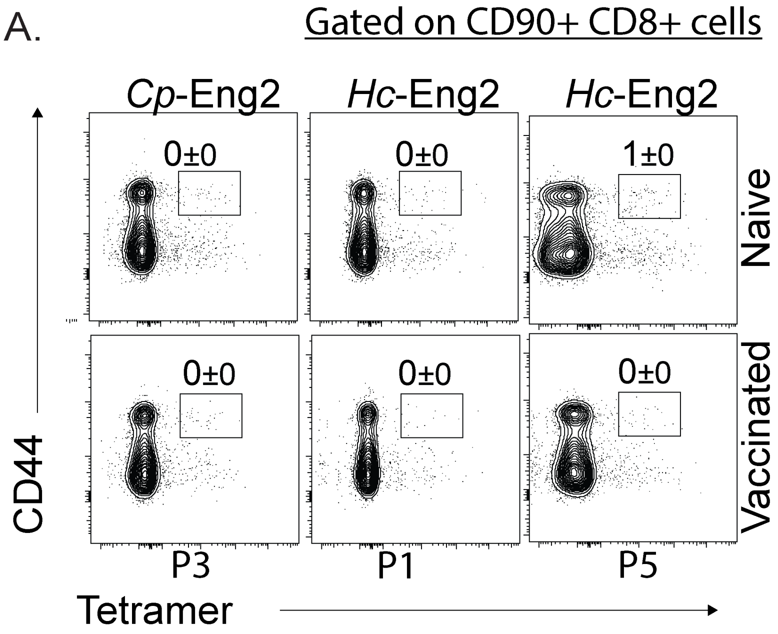

B.

Tetramer	Naïve(Precursor)	Vaccinated	Fold expansion
Cp-Eng2 (P3)	130	1292	7
Hc-Eng2 (P1)	47	357	11
Hc-Eng2 (P5)	32	330	29
Bl-Eng2 (P1)	97	839	11

C.

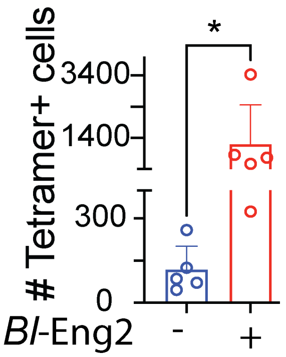

Supplementary figure 3 related to figure 6

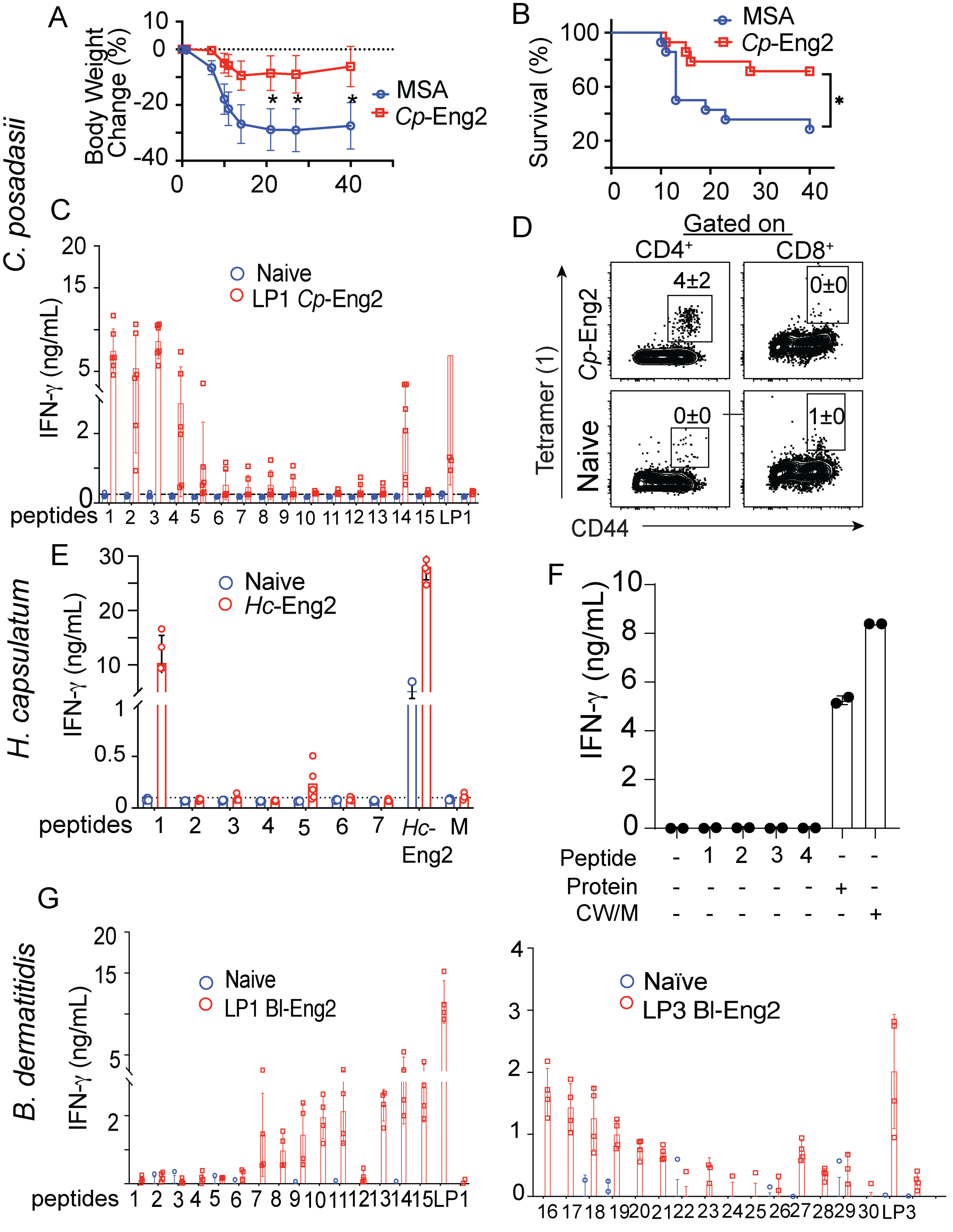

Supplementary figure 4 related to Figure 6

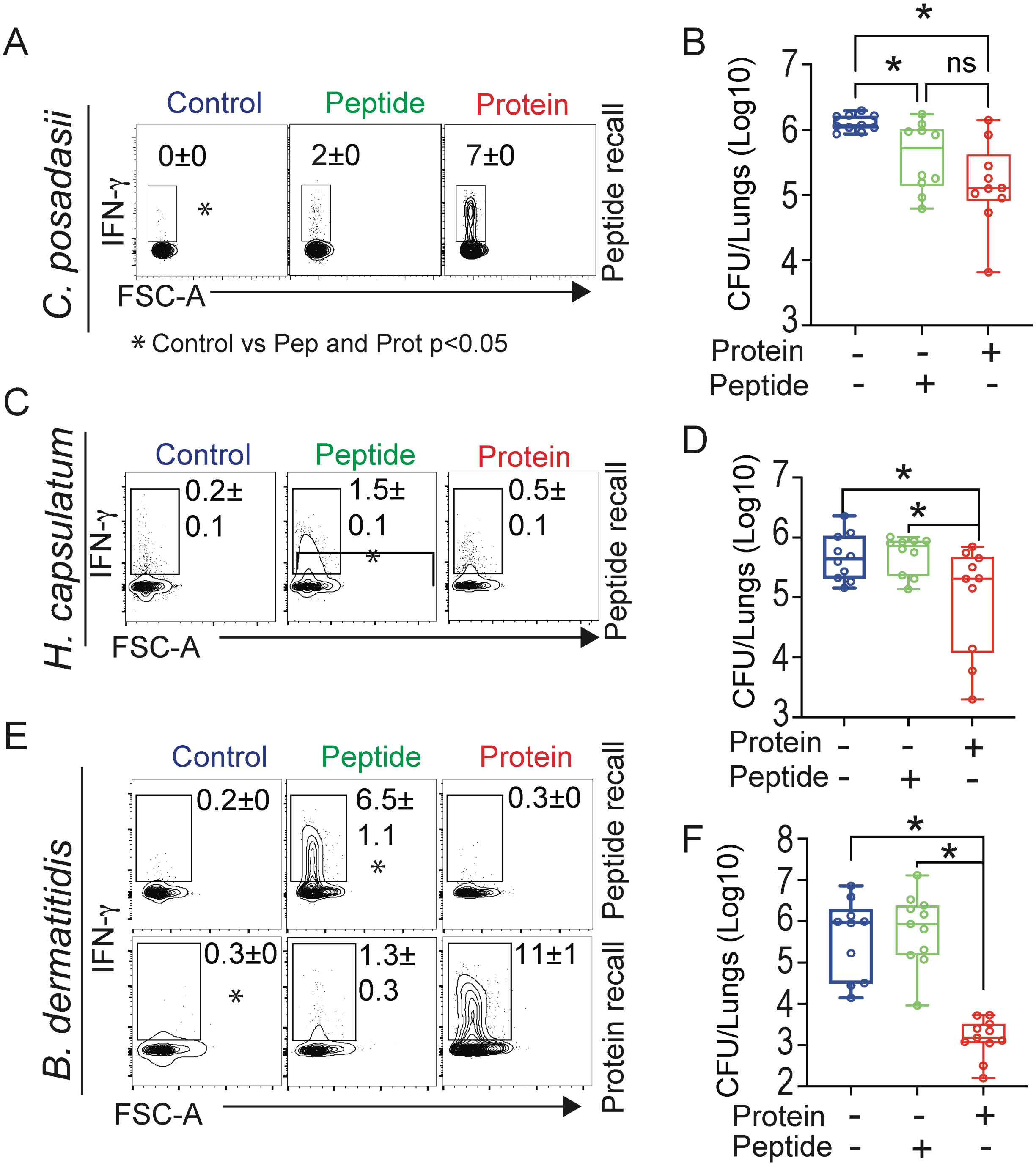

Supplementary Figure 5 related to Methods

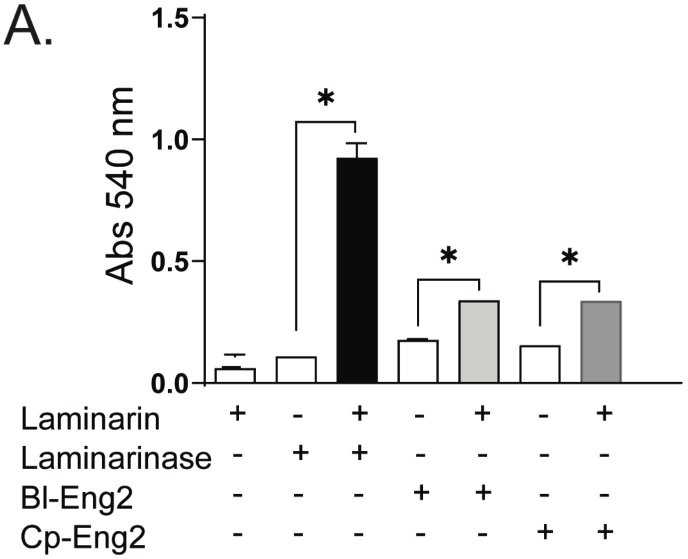

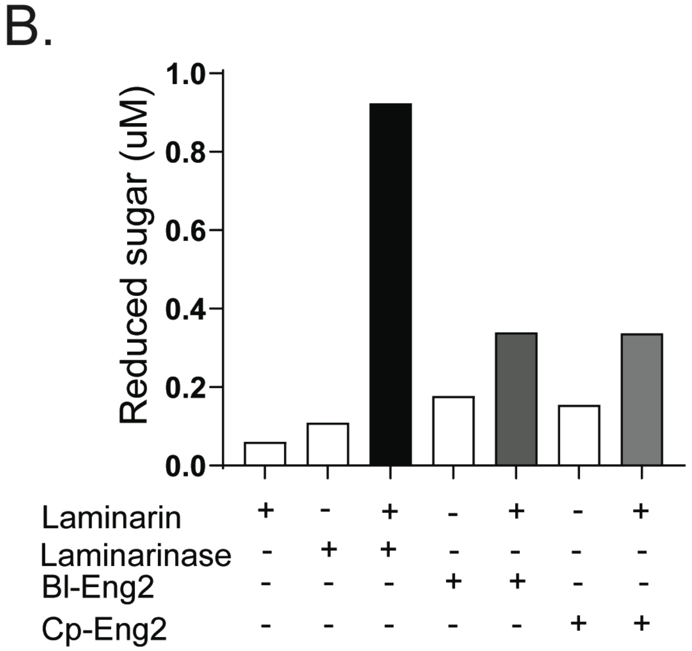

Supplementary Table 1. Peptides used to stimulate cells in healthy donors and patients with infection (related to main Figures 7 and 8)

Sequence source	Peptide sequence	Peptide length
***Bl*-Eng2**		
P1	ATEWTSDSISVWFFPRYQIPSNINDENPD	29
P2	CEFDKFFQEQRIIFNTAFCGDWAKAT	26
P3	KNNPWAFSEAFWSINYMKVFQNKQGD	26
***Cp*-Eng2**		
P1	GVYAMEWTSDEITVWFFPRGNIPDDVNSQ	29
P2	GDCDLDRFVQDQRIIFNTAFCGDWAKGLW	29
P3	KNNPKDFAEAYWEIYGMKVYSKGQGQKI	28
P4	MPGSICGVWPAFWTVGSRWPEHGEMDI	27
***Hc*-Eng2**		
P1	EWTTDSISVWFFPRYRIPSDINSEHPD	27
P2	EFDKFFQEQRIIINTAFCGDWA	22
P3	QPSNFFDNFNFFNGPDPSNGYVT	20
P4	YWSINYMKVFQDEVVDYPGD	18
